# Decoding the Genetic Puzzle of Inherited Retinal Dystrophies: Novel Insights From a Turkish Cohort

**DOI:** 10.1111/cge.14769

**Published:** 2025-05-15

**Authors:** Şenol Demir, Esra Arslan Ateş, Orkun Sevik, Bengisu Sözer, Tuğba Köse, Özlem Şahin, Ahmet Arman, Bilgen Bilge Geçkinli

**Affiliations:** ^1^ Department of Medical Genetics School of Medicine, Marmara University Istanbul Turkey; ^2^ Department of Medical Genetics Istanbul University‐Cerrahpaşa, Cerrahpaşa Faculty of Medicine Istanbul Turkey; ^3^ Department of Ophthalmology School of Medicine, Marmara University Istanbul Turkey; ^4^ Department of Genetics and Bioengineering Faculty of Engineering and Natural Sciences, Istanbul Bilgi University Istanbul Turkey

**Keywords:** inherited retinal dystrophies, IRDs, next‐generation sequencing, retinitis pigmentosa

## Abstract

Inherited retinal dystrophies (IRDs) are genetic disorders characterized by retinal pigment epithelium or photoreceptor degeneration. Advances in molecular diagnostic technologies, particularly next‐generation sequencing (NGS), have facilitated the identification of disease‐causing variants; however, population‐specific genetic data, especially for Turkish cohorts, remain limited. This study aims to investigate the genetic profile of IRD patients in a Turkish cohort and assess the diagnostic utility of NGS‐based gene panel testing. A total of 94 patients diagnosed with IRDs were included in the study. Genomic DNA was extracted from the peripheral blood of patients who met the inclusion and exclusion criteria. NGS was performed to analyze 141 genes associated with IRDs, following current clinical guidelines and utilizing up‐to‐date variant databases. Among the 94 patients, 97 variants were identified in 70 patients (74%). Of these, 58 variants (59.79%) were classified as pathogenic and 39 variants (40.21%) as likely pathogenic. Additionally, 28 variants (28%) were novel and have not been previously reported in the literature. Our findings demonstrate that NGS is a powerful tool for the molecular diagnosis of IRDs and emphasizes the genetic diversity of IRDs in the Turkish population. The identification of novel variants also highlights the need for continued variant curation and population‐specific studies to enhance diagnostic accuracy and genetic counseling.

## Graphical abstract

This study analyzes 94 IRD patients from a Turkish cohort using a 141‐gene NGS panel, achieving a 74% diagnostic yield. The identification of 28 novel variants highlights the genetic diversity of IRDs in Türkiye and underscores the value of population‐specific molecular testing.

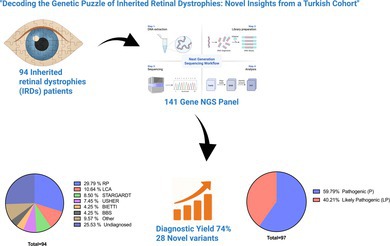

## 1 Introduction

Inherited retinal dystrophies (IRDs) are a group of progressive genetic disorders characterized by retinal pigment epithelium or photoreceptor degeneration. The prevalence is approximately 1 in 3000 [1]. IRD represents a highly heterogeneous group of disorders, both clinically and genetically.

The specific cell type involved in degeneration varies across subtypes. In many cases, retinal pigment epithelium (RPE) cells are primarily affected, while in others, photoreceptors are more directly impacted. When rod cells are predominantly affected, as in retinitis pigmentosa (RP), patients typically experience night blindness and a reduction in peripheral visual fields. In contrast, subtypes that mainly involve cone cells, such as cone dystrophies or macular dystrophies, are characterized by central vision loss. As the disease progresses, both rod and cone cells, along with RPE cells, may become involved in the degenerative process. The inheritance pattern of IRDs varies among related genes. To date, 281 genes associated with hereditary retinal dystrophies have been reported in the current database Retinal Information Network (RETNET) (https://sph.uth.edu/retnet/). Autosomal recessive (AR), autosomal dominant (AD), X‐linked inheritance, or mitochondrial inheritance can be observed. Especially in Türkiye, where consanguineous marriages are frequent, AR types are more common, but other inheritance types can also be seen. In addition, IRDs may be isolated or may be one of the components of a syndrome [2]. Among these syndromes are Usher Syndrome (US), Bardet‐Biedl Syndrome (BBS), Joubert Syndrome, and Alstrom Syndrome. Molecular genetic analyses are very important at this stage, as it is very difficult to determine the disease subtype based on clinical findings alone.

This study aimed to investigate the diagnostic yield of a 141‐gene panel applied by next‐generation sequencing (NGS) in the Turkish population and correlate the clinical and genetic findings. We also aimed to explore how effectively this approach identifies pathogenic variants and contributes to expanding the mutational spectrum in this population.

## 2 Materials and Methods

### 2.1 Cohort Description

The study included 94 Turkish‐origin patients who were clinically diagnosed with IRDs. Clinical diagnosis of IRDs in the study cohort was established by a multidisciplinary team of ophthalmologists and clinical geneticists. Diagnostic criteria were based on detailed ophthalmologic evaluations, including visual acuity, fundus examination, optical coherence tomography (OCT), and full‐field electroretinography (ffERG), combined with medical history and pedigree analysis. Patients included in the study were diagnosed with IRDs based on clinical and genetic findings. The classification of patients into syndromic and non‐syndromic cases was determined based on the presence or absence of extraocular manifestations or involvement of other systems.

Syndromic IRDs were defined as cases where retinal dystrophy coexists with other systemic features, such as those seen in Usher syndrome, Bardet‐Biedl syndrome, or Alström syndrome. Non‐syndromic IRDs referred to cases where retinal dystrophy was the primary manifestation, without additional systemic involvement.

For this research, the Ethics Committee of Marmara University approval was obtained with the decision of the Board of Ethics Committee numbered 09.2023.876. Also, All participants (or their legal guardians) provided written informed consent before genetic testing and inclusion in the study.

### 2.2 DNA Isolation and Next Generation Sequencing

A 5 mL whole blood sample was collected in blood tubes containing ethylene diamine tetra acetic acid (EDTA), “Lab‐Aid 824 DNA Isolation Kit” (Xiamen Zeesan Biotech Co. Ltd., P.R. China) and “LabAid 824s Nucleic Acid Isolator” (Xiamen Zeesan Biotech Co. Ltd., P.R. China) were used for DNA isolation. The purity and concentration of DNA samples were measured using a Qubit 3.0 Fluorometer (Thermo Fisher Scientific, Waltham, MA). A260/A280 and A260/A230 ratios, two parameters indicating the purity of DNA, were measured. These two ratios should be between 1.8 and 2.2 for optimal DNA purity. DNA samples meeting these criteria and having an initial concentration of at least 100 ng in 15 μL were included in the study.

Clinical Exome Sequencing (CES) was performed using the NGS‐based Clinical Exome Solution v3 kit (SOPHiA Genetics, SA, Switzerland) covering the exons and exon‐intron junction regions of 6380 genes. NextSeq550DX NGS (Illumina) instrument was used for the NGS sequencing protocol. The data obtained after sequencing were analyzed on the Sophia DDM (Sophia Genetics SA, Switzerland) platform. A virtual panel containing 141 IRD‐related genes was created. The panel used in this study was designed to include 141 genes known to be associated with inherited retinal dystrophies (IRDs), based on current literature, clinical relevance, and gene‐disease associations listed in the RetNet database (https://sph.uth.edu/retnet) (Table S1). The following filtering methods were applied for the pathogenicity evaluation of the variants obtained:

1. Variants with variant fraction between 15% and 100% were analyzed.
2. Variants with minor allele frequencies below 0.1% according to the genome assembly database (gnomAD), 1000 genomes (1000G) (1000 Genomes Project Consortium, 2012) and exome sequencing project (ESP5400) (NHLBI GO Exome Sequencing Project) databases were selected.
3. Variants reported in the ClinVar database as “Benign” and/or “Likely benign” were excluded.
4. Synonymous variants that were predicted not to affect splicing according to “Mutation Taster” and “Human Splicing Finder (HSF)” and intronic variants within 20 base pairs of the splice site were eliminated by filtering [3, 4].
5. All missense, nonsense, frameshift, inframe deletion/duplications, and 20 base pair of the exon‐intron junction region intronic variants were selected.
6. The pathogenicity of the variants remaining after the filtering process was evaluated using ACMG criteria [5]. For this purpose, the Varsome program, which gives pathogenicity scores using ACMG criteria, was used [6]. Variants evaluated as “benign” and/or “possible benign” according to these criteria were eliminated.
7. The remaining variants were evaluated with the patient's clinic and segregation studies, and clinically relevant variants were reported.

Segregation analyses were performed to confirm the variations detected in the index cases obtained after CES analyses and to determine the presence of the variant detected in first‐degree relatives such as mother, father, siblings, and relatives with a similar history, and to show the distribution within the family. PCR (polymerase chain reaction) was done using forward and reverse primers appropriate for the exons having the identified variants on a Biorad CFX96 Touch thermal cycler (Biorad; Berkeley, CA, USA). Sequencing was performed with ABI PRISM 3130xl Genetic Analyzer (Applied Biosystems, United States of America). Data analysis was performed with Chromas (Technelysium Pty Ltd., Australia) analysis program.

### 2.3 Statistical Analysis

All statistical analyses and charts were presented using GraphPad Prism, version 9.5.0. Descriptive statistical methods (mean, standard deviation, frequency, and ratio) were used to summarize the data. Results were intended to offer a general overview of the study group. To prevent misinterpretation of percentages in subgroups with small sample sizes, the number of observations (*n*) was reported in addition to percentages in all relevant figures and tables.

Demographic and clinical features (age, age at onset of visual symptoms, sex, consanguinity, family history, and extraocular findings) were summarized using appropriate descriptive statistics. Genetic variants were categorized by type and pathogenicity, and their distributions were shown as percentages.

## 3 Results

The mean age of the patients (1–80 years) was 29.03 ± 17.98 years; 52 of them were male (55.32%), and 42 were female (44.68%) (Table 1). The mean age at the onset of visual complaints of the patients included in the study was 15.29 ± 13.74 years. However, for undiagnosed patients, the age at the onset of complaints was 22.71 ± 19.09 years. Forty patients had a family history (42.55%) of retinal dystrophy. This rate was 50% in diagnosed patients and 20% in undiagnosed patients. In addition, 42 of the patients had findings affecting other systems accompanying ocular findings (44.68%). Among the diagnosed patients, 28 were diagnosed as isolated RP (40%), and 42 were diagnosed as syndromic RP (60%). Consanguineous marriage between parents was present in 69 (73%) of the patients included in the study. Among the 18 undiagnosed cases, eight patients (44%) had consanguinity between their parents.

**TABLE 1:** Demographic data and clinical information.

	RP (*n* = 28)	LCA (*n* = 10)	Bietti (*n* = 4)	Usher (*n* = 7)	STARGARDT (*n* = 8)	BBS (*n* = 4)	Other (*n* = 9)	Undiagnosed (*n* = 24)	Total (*n* = 94)
Age (mean ± SD)	34.07 ± 16.41	15.70 ± 12.42	27.25 ± 11.79	31.86 ± 14.96	19.50 ± 10.57	17.25 ± 3.30	17.44 ± 10.53	37.67 ± 21.99	29.03 ± 17.98
Age of onset of visual complaints (mean ± SD)	16.00 ± 11.92	4.60 ± 8.13	13.25 ± 3.86	18.71 ± 08.90	12.38 ± 04.37	11.75 ± 9.77	7.55 ± 8.52	22.71 ± 19.09	15.29 ± 13.74
Gender (%)									
Female	53.58	50.00	75.00	57.14	62.50	25.00	44.44	20.84	44.68
Male	46.42	50.00	25.00	42.86	37.50	75.00	55.56	79.16	55.32
Consanguineous marriage (%)									
Yes	82.14	90.00	100.00	85.71	62.50	75.00	100.00	41.67	73.40
No	17.86	10.00	0.00	14.29	37.50	25.00	0.00	58.33	26.60
Family history (%)									
Yes	53.58	40.00	50.00	71.43	62.50	25.00	33.33	20.83	42.55
No	46.42	60.00	50.00	28.57	37.50	75.00	66.67	79.17	57.45
Extraocular finding (%)									
Yes	25.00	30.00	25.00	100.00	14.29	100.00	66.67	54.17	44.68
No	75.00	70.00	75.00	0.00	85.71	0.00	33.33	45.83	55.32

*Note*: The number of samples of each group is indicated by *n*. The total number of samples was 94. Diseases evaluated within the scope of “Other”; PHARC, Oguchi, Retinal cone dystrophy 3b, Aniridia, Achromotopsia, Alstrom, Achromatopsia, Retinoschisis, Gyrate atrophy.

Abbreviations: BBS, Bardet Biedl syndrome; Bietti, Bietti crystalline corneoretinal dystrophy; LCA, Leberin congenital amaurosis; Other, includes one patient from each of the following clinical conditions; RP, retinitis pigmentosa; STARGARDT, Stargardt's disease; Usher, Usher syndrome.

**FIGURE 1:**
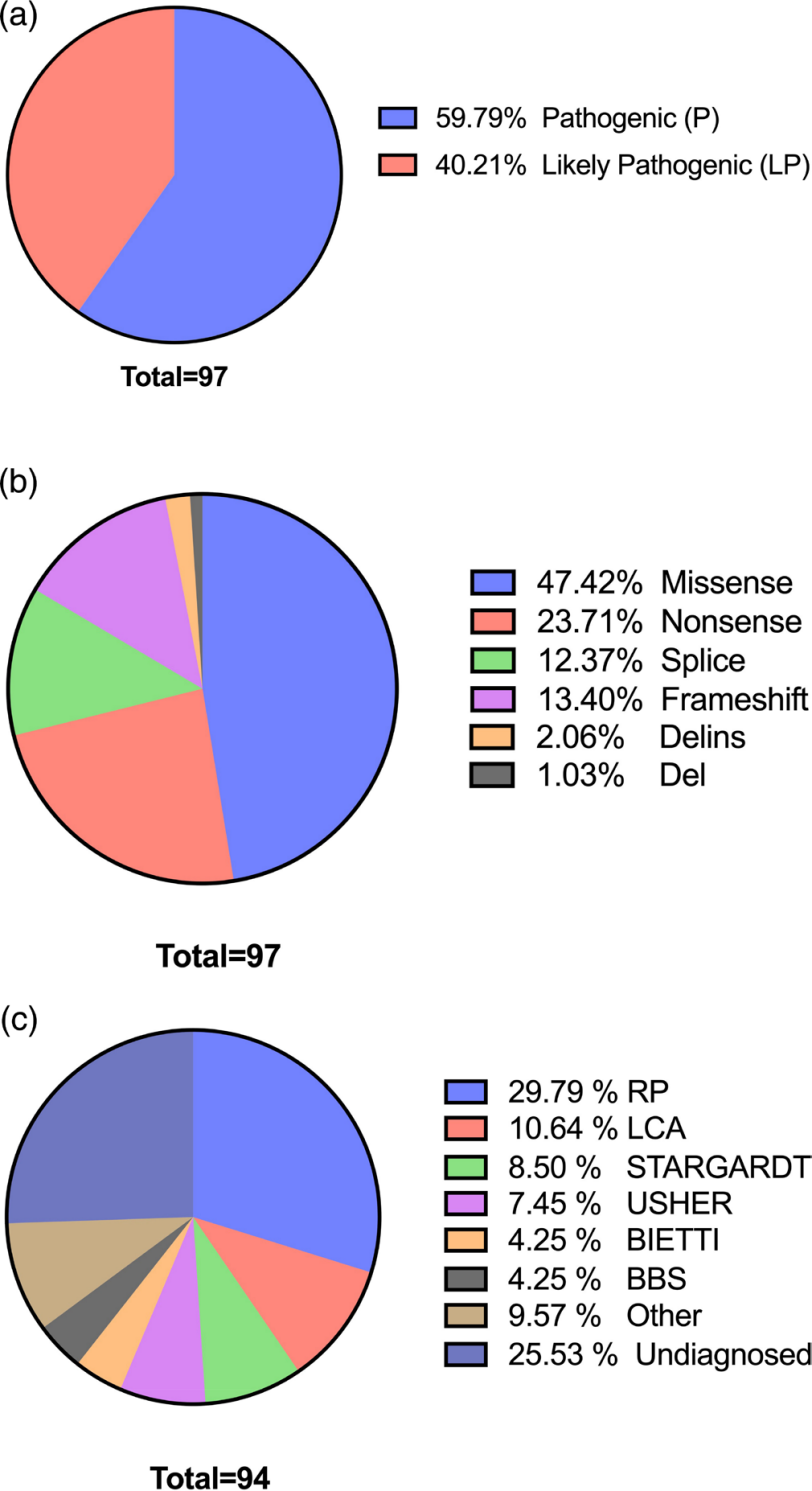
(a) Pathogenicity classification of variants. (b) Types of detected variants. (c) Distribution of patient ratios according to IRD subtypes.

A total of 97 variants in 45 distinct genes were detected in 70 (74%) patients, of which 58 (59.79%) were pathogenic, 39 (40.21%) were likely pathogenic (Table 2) (Figure 1a). Of these variants, 46 (47.42%) were missense, 23 (23.71%) were nonsense, 12 (12.37%) were splice site, 13 (13.40%) were frameshift, 2 (2.06%) were deletion insertion (delins) and 1 (1.03%) was deletion (Figure 1b). These variants were identified in genes associated with specific IRDs, confirming clinical and genetic diagnoses in the following cases: isolated retinitis pigmentosa (RP) in 28 patients (29.79%), Leber congenital amaurosis (LCA) in 10 patients (10.64%), Stargardt disease in 8 patients (8.50%), Usher syndrome (US) in 7 patients (7.45%), Bietti crystalline dystrophy (BCD) in 4 patients (4.25%), and Bardet‐Biedl syndrome (BBS) in 4 patients (4.25%) (Figure 1c). Additionally, 9 patients (9.57%) were found to have rare genetic subtypes of IRDs, classified under the ‘other’ category as indicated in the table (Table 1).

**TABLE 2:** Pathogenicity of detected variants in patients.

Patient	Gene	Variant	Protein alteration	Zygosity	DANN score	ACMG criteria	Pathogenicity	Mode of inheritance
P1	ABCA4	c.3244G>T	(p.Val1082Leu)	Heterozygous	1.0	PM1 PP2 PM2 PP3	LP	AR
P2	USH2A	c.12067–2A>G	(p.?)	Homozygous	0.99	PVS1 PM2 PM3 PP1	P	AR
P2	ABCA4	c.5882G>A	(p.Gly1961Glu)	Heterozygous	1.0	PS3 PM2 PM5 PP3 PP2 PP5	P	AR
P3	PROM1	c.2023C>T	(p.Arg675*)	Homozygous	0.98	PS4 PVS1 PM2	P	AR
P3	PRPF8	c.4980C>G	(p.Tyr1660*)	Heterozygous	1.0	PVS1 PM2	LP	AD
P7	USH2A	c.12067–2A>G	(p.?)	Heterozygous	0.99	PVS1 PM2 PM3 PP1	LP	AR
P7	USH2A	c.6485A>C	p.Gln2162Pro	Heterozygous		PM2 PP4	LP	AR
P8	ABHD12	c.871delT	(p.Tyr291Ilefs*28)	Homozygous		PVS1 PM2	LP	AR
P10	CRB1	c.2786T>C	(p.Met929Thr)	Heterozygous	0.95	PM3 PM2 PM5 PM1 PP2 PP5	P	AR
P10	TTC8	c.9_10dupGG	(p.Glu4Glyfs*41)	Homozygous		PVS1 PM2	LP	AR
P11	BBS1	c.479G>A	(p.Arg160Gln)	Homozygous	1.0	PM3 PM2 PP3 PP2 PP5	P	AR
P12	CYP4V2	c.414–1G>T	(p.?)	Homozygous	1.0	PVS1 PM2	LP	AR
P14	CYP4V2	c.802–8_810delinsGC	(p.?)	Homozygous		PM3 PVS1 PM2 PP5	P	AR
P15	RP1	c.269del	(p.His90Profs*26)	Homozygous		PVS1 PM2 PS4 PP5	P	AD, AR
P16	CRB1	c.2230C>T	(p.Arg744*)	Homozygous	1.0	PVS1 PM2 PS4 PP5	P	AR
P17	RDH12	c.295C>A	(p.Leu99Ile)	Homozygous	1.0	PM3 PM2 PP3 PP2 PS3 PP1 PP5	P	AD, AR
P18	CYP4V2	c.802–8_810delinsGC	(p.?)	Homozygous		PM3 PVS1 PM2 PP5	P	AR
P19	USH2A	c.11864G>A	(p.Trp3955*)	Heterozygous	0.99	PVS1 PM3 PM2 BS2 PP5	P	AR
P19	USH2A	c.2276G>T	(p.Cys759Phe)	Heterozygous	0.99	PM3 PP1 PM2 PS4 PP3 PM1 PP4 PP5	LP	AR
P20	MAK	c.668_671del	(p.Asp223Glyfs*11)	Homozygous		PVS1 PM2 PP5	P	AR
P21	RDH12	c.444C>A	(p.His148Gln)	Homozygous	0.99	PM2 PM1 PP3 PP2 PP5	LP	AD, AR
P22	ABCA4	c.5882G>A	(p.Gly1961Glu)	Heterozygous	1.0	PS3 PM2 PM5 PP3 PP2 PP5	P	AR
P22	ABCA4	c.3808G>T	(p.Glu1270*)	Heterozygous	1.0	PVS1 PM2 PS4 PP5	P	AR
P23	CYP4V2	c.1198C>T	(p.Arg400Cys)	Homozygous	0.99	PM3 PM2 PM5 PP3 PM1 PS2 PP5	P	AR
P24	CRB1	c.1982C>A	(p.Ala661Glu)	Homozygous	0.96	PM2 PP2 PP4	LP	AR
P25	GRK1	c.1610_1613del	(p.Asp537Valfs*7)	Homozygous		PM3 PM2 PVS1 PP5	P	AR
P26	ABCA4	c.5882G>A	(p.Gly1961Glu)	Heterozygous	1.0	PS3 PM2 PM5 PP3 PP2 PP5	P	AR
P26	ABCA4	c.2288C>A	(p.Ala763Asp)	Heterozygous	1.0	PS4 PM2 PM5 PM1 PP3 PP2 PP5	P	AR
P28	ABCA4	c.203C>T	(p.Pro68Leu)	Heterozygous	1.0	PS4 PP3 PM2 PM5 PM1 PP2 PP5	P	AR
P28	ABCA4	c.2966T>A	(p.Val989Asp)	Heterozygous	0.99	PM2 PM5 PM1 PP3 PP2	LP	AR
P29	ABCA4	c.3749T>C	(p.Leu1250Pro)	Homozygous	1.0	PP3 PS4 PM2 PP2 PP5	P	AR
P30	ABCA4	c.3808G>T	(p.Glu1270*)	Heterozygous	1.0	PVS1 PM2 PS4 PP5	P	AR
P31	KCNV2	c.1356+3_1356+6del	(p.?)	Homozygous		PP3 PM2 PM3 PP4 PP5	LP	AR
P35	PAX6	c.467G>A	(p.Trp156*)	Heterozygous	1.0	PVS1 PM2 PS4 PP5	P	AD
P36	CNGB3	c.1006G>T	(p.Glu336*)	Heterozygous	1.0	PVS1 PM3 PM2 PP5	P	AR
P37	KCNJ13	c.500T>A	(p.Ile167Asn)	Homozygous	0.99	PP3 PM2 PP4	LP	AR
P38	CRB1	c.1894C>T	(p.Arg632*)	Homozygous	1.0	PVS1 PM2 PS4 PP5	P	AR
P38	PDE6B	c.2399del	(p.Leu800Argfs*19)	Heterozygous		PVS1 PM2	LP	AR
P39	PROM1	c.1090C>T	(p.Arg364Cys)	Heterozygous	0.92	PS4 PM2 PP5	LP	AD, AR
P40	CNGA1	c.947C>T	(p.Ser316Phe)	Homozygous	1.0	PM3 PP3 PM2 PS3 PP1 PP5	P	AR
P40	POMGNT1	c.1462C>T	(p.Arg488*)	Heterozygous	1.0	PVS1 PM3 PM2 PP5	P	AR
P41	PCARE	c.958del	(p.Arg320Alafs*3)	Homozygous		PVS1 PM3 PM2 PP5	P	AR
P42	ABCA4	c.5315G>A	(p.Trp1772*)	Homozygous	1.0	PVS1 PM2 PS4 PP5	P	AR
P43	TULP1	c.888T>G	(p.Asn296Lys)	Homozygous	1.0	PM3 PM2 PP3 PM1 PP5	LP	AR
P44	BBS4	c.587A>G	(p.Asp196Gly)	Homozygous	1.0	PP5 PP3 PM2	LP	AR
P44	USH2A	c.12569T>C	(p.Val4190Ala)	Heterozygous	1.0	PM3 PM2 PM1 PP1 PP5	LP	AR
P47	ALMS1	c.7313C>A	(p.Ser2438*)	Homozygous	1.0	PVS1 PM3 PM2 PP1 PP5	P	AR
P47	RHO	c.520G>A	(p.Gly174Ser)	Heterozygous	1.0	PP3 PM2 PP2	P	AD, AR
P48	USH2A	c.11156G>A	(p.Arg3719His)	Homozygous	1.0	PM3 PM2 PM5 PP1 PP5	P	AR
P48	ABCA4	c.5882G>A	(p.Gly1961Glu)	Heterozygous	1.0	PS3 PM2 PM5 PP3 PP2 PP5	P	AR
P49	IFT172	c.3228+13G>A	(p.?)	Homozygous		PP4 PM2 BP7	LP	AR
P50	CERKL	c.316C>A	(p.Arg106Ser)	Homozygous	1.0	PM3 PM2 PM5 PP5	P	AR
P50	PDE6B	c.1108–3C>G	(p.?)	Heterozygous		PP3 PM2 PP4	LP	AR
P51	RDH12	c.379G>T	p.Gly127*	Homozygous	0.99	PVS1 PM3 PM2 PP5	P	AD, AR
P51	ABCA4	c.5882G>A	(p.Gly1961Glu)	Heterozygous	1.0	PS3 PM2 PM5 PP3 PP2 PP5	P	AR
P52	USH2A	c.6127_6128dup	(p.Ser2043ArgfsTer6)	Homozygous		PVS1 PM3 PM2 PP5	P	AR
P53	CNGB1	c.413–1G>A	(p.?)	Homozygous	0.95	PVS1 PM3 PM2 PP5	P	AR
P55	ABCA4	c.1554+3_1554+4del	(p.?)	Homozygous		PM2 PP4	LP	AR
P56	MYO7A	c.6539T>C	(p.Leu2180Pro)	Homozygous	1.0	PP4 PP3 PM2	LP	AR
P59	BBS7	c.849+1G>T	(p.?)	Homozygous	1.0	PVS1 PM2	LP	AR
P62	CNGB3	c.1179–2A>T	(p.?)	Homozygous	0.99	PVS1 PM3 PM2 PP5	P	AR
P62	CNGB1	c.2629G>A	(p.Gly877Arg)	Heterozygous	1.0	PP3 PM3 PM2 PP5	P	AR
P63	USH2A	c.11156G>A	(p.Arg3719His)	Heterozygous	1.0	PM3 PM2 PM5 PP1 PP5	P	AR
P64	ALMS1	c.3154C>T	(p.Gln1052*)	Homozygous	0.99	PVS1 PM2	LP	AR
P65	RS1	c.52+1G>C	(p.?)	Hemizygous	0.99	PVS1 PM2	LP	XLR
P66	PRPH2	c.461_464delinsTGGTCT	(p.Lys154Metfs*103)	Homozygous		PVS1 PM2 PS4 PP5	P	AD, AR
P67	TULP1	c.1199G>T	(p.Arg400Leu)	Homozygous	1.0	PM2 PM5 PM1 PP3	LP	AR
P69	ABCA4	c.5172G>A	(p.Trp1724*)	Heterozygous	1.0	PVS1 PM2 PS4 PP5	P	AR
P69	ABCA4	c.5882G>A	(p.Gly1961Glu)	Heterozygous	1.0	PS3 PM2 PM5 PP3 PP2 PP5	P	AR
P70	ABCA4	c.5909T>C	(p.Leu1970Pro)	Homozygous	1.0	PS4 PP3 PM2 PM5 PM1 PP2 PP5	P	AR
P72	ARL6	c.276del	(p.Phe92Leufs*9)	Homozygous		PVS1 PM2	LP	AR
P73	PDE6A	c.1166C>T	(p.Pro389Leu)	Homozygous	1.0	PP3 PM2 PP5	LP	AR
P74	BEST1	c.1013G>A	(p.Trp338*)	Homozygous	1.0	PVS1 PM2 PS4 PP5	P	AR
P75	RHO	c.491C>T	(p.Ala164Val)	Heterozygous	1.0	PS4 PM2 PM5 PM1 PP2 PP5	P	AD, AR
P75	TULP1	c.1082G>A	(p.Arg361Gln)	Heterozygous	1.0	PM2 PP5 PP3 PM1	LP	AR
P76	RHO	c.403C>T	(p.Arg135Trp)	Heterozygous	1.0	PP1 PS2 PM2 PM5 PM1 PP3 PP2	P	AD, AR
P77	PRPH2	c.470A>G	(p.Asp157Gly)	Heterozygous	1.0	PS4 PP3 PM2 PM5 PM1 PP2 PP5	P	AD, AR
P77	BBS2	c.1015C>T	(p.Arg339*)	Heterozygous	1.0	PVS1 PM3 PM2 PP5	P	AR
P78	USH2A	c.12574C>G	(p.Arg 4192Gly)	Homozygous	0.99	PM2 PM5 PM1	LP	AR
P78	ABCA4	c.3149G>A	(p.Gly1050Asp)	Heterozygous	1.0	PM3 PM2 PM1 PP1 PP5 PM5	P	AR
P79	PDE6B	c.169_239dup	(p.Leu83Cysfs*91)	Homozygous		PVS1 PM2 PP5	P	AR
P80	MERTK	c.1744_1751delinsT	(p.Ile582*)	Homozygous		PVS1 PM3 PM2 PP5	P	AR
P81	PROM1	c.2118G>T	(p.Gly706Gly)	Homozygous		PM2 PP3 PP4	LP	AR
P82	ABCA4	c.571–2A>T	(p.?)	Homozygous	0.99	PVS1 PS4 PM2 PP5	P	AR
P83	EYS	c.1185–3C>A	(p.?)	Homozygous	N	PM2 PP3	LP	AR
P84	RP1	c.109C>T	(p.Arg37*)	Homozygous	1.0	PVS1 PM2	LP	AD, AR
P84	HMCN1	c.12095G>A	(p.Gly4032Asp)	Homozygous	0.99	PM2 PP3	LP	AD
P85	SCLT1	c.37C>T	(p.Arg13*)	Heterozygous	1.0	PVS1 PM2 PP5	P	AR
P86	MYO7A	c.4793T>C	(p.Leu1598Pro)	Homozygous	1.0	PP3 PM2	LP	AR
P87	LCA5	c.103C>T	(p.Arg35*)	Homozygous	1.0	PVS1 PM3 PM2 PP5	P	AR
P88	OAT	c.748C>T	(p.Arg250*)	Homozygous	1.0	PVS1 PM3 PM2 PP5	P	AR
P89	IMPG2	c.1491del	(p.Leu498Cysfs*15)	Homozygous		PVS1 PM2 PS4 PP5	P	AR
P90	PDE6B	c.1935C>G	(p.Tyr645*)	Homozygous	1.0	PVS1 PM2	LP	AR
P91	ABCA4	c.286A>G	(p.Asn96Asp)	Heterozygous	1.0	PM3 PM2 PM5 PM1 PP5 PP3 PP2	P	AR
P92	IMPDH1	c.809T>C	(p.Leu270Pro)	Homozygous	1.0	PP3 PM2 PM5	LP	AD
P93	LRAT	c.481T>C	(p.Cys161Arg)	Homozygous	1.0	PM2 PP3 PM1 PM3 PP5	LP	AR
P94	PDE6B	c.243del	(p.Arg82Alafs*68)	Homozygous		PVS1 PM2	LP	AR

Abbreviations: AD, autosomal dominant; AR, autosomal recessive; LP, likely pathogenic; N/A, not available; P, pathogenic.

## 4 Discussion

**FIGURE 2:**
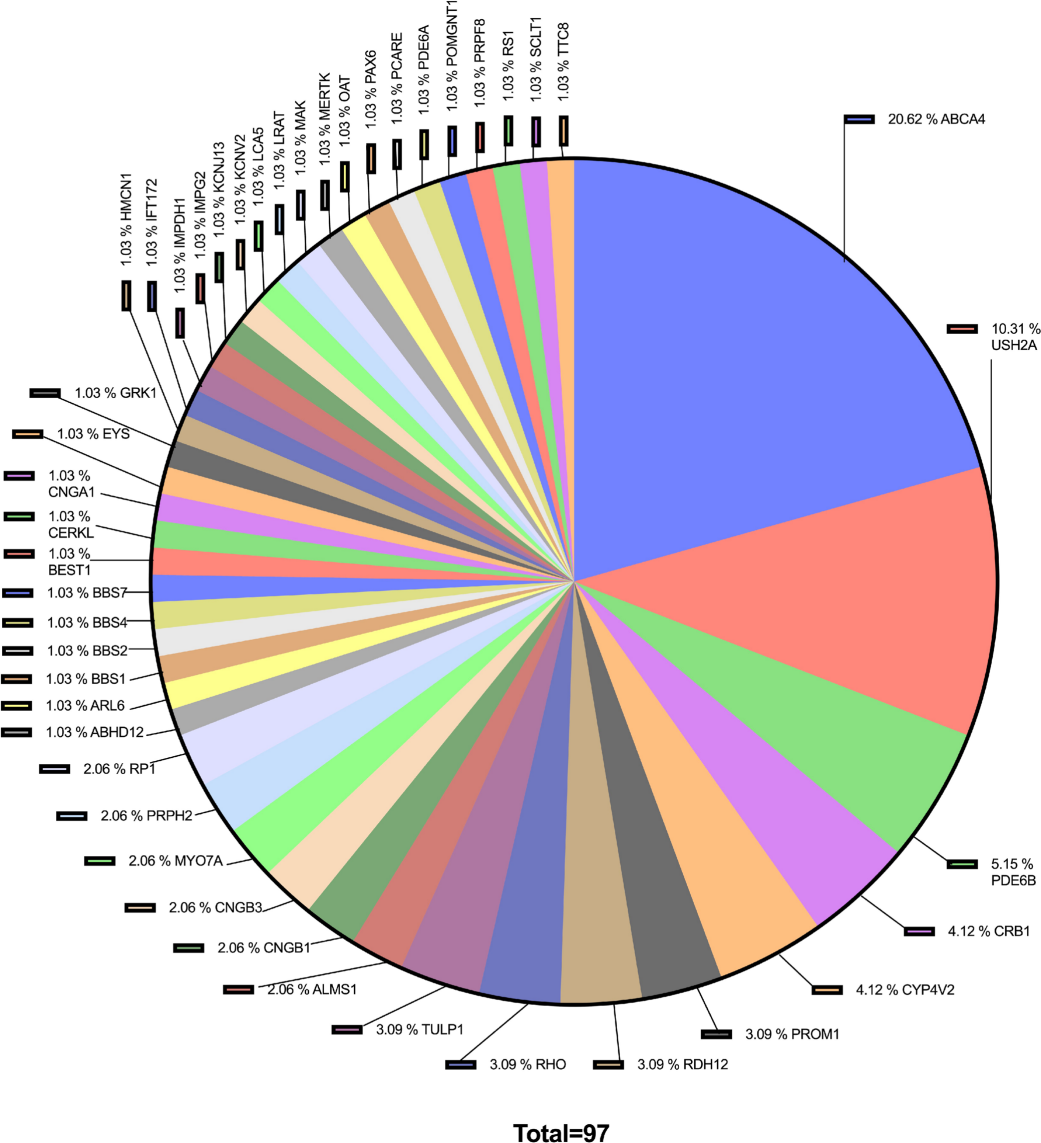
Distribution of variants detected in patients according to genes.

Molecular analysis of IRDs patients contributes to our understanding of the genetic and molecular basis of this clinically and genetically heterogeneous disease group. In our study, a molecular diagnosis was established in 70 (74%) of the 94 patients studied. Diagnosis rates have been reported to be between 70% and 80% in recent studies in the literature [7, 8]. In the 94 patients included in the study, 97 variants were found. Of these, 28 (29%) variants were reported for the first time in this study (Table 3). The most common variant was found in the *ABCA4* gene (20 variants in 16 patients) which is similar to the cohort studies [9, 10] (Figure 2).

**TABLE 3:** List of detected novel variants.

Patient	Gene	Transcript number	c.DNA	Amino acid change	Zygosity	DANN score	Classification
P1	ABCA4	NM:000350.3	c.3244G>T	p.Val1082Leu	Heterozygous	0.9979	LP
P3	PRPF8	NM:006445	c.4980C>G	p.Tyr1660*	Heterozygous	0.9958	LP
P7	USH2A	NM:206933	c.6485A>C	p.Gln2162Pro	Heterozygous	0.9957	LP
P8	ABHD12	NM:001042472	c.871delT	p.Tyr291Ilefs*28	Homozygous	N/A	LP
P10	TTC8	NM:144596.4	c.9_10dupGG	p.Glu4Glyfs*41	Homozygous	N/A	LP
P12	CYP4V2	NM:207352	c.414‐1G>T	—	Homozygous	0.995	LP
P24	CRB1	NM:001193640	c.1982C>A	p.Ala661Glu	Homozygous	0.9631	LP
P28	ABCA4	NM:000350.3	c.2966T>A	p.Val989Asp	Heterozygous	0.9867	LP
P37	KCNJ13	NM:001172417	c.500T>A	p.Ile167Asn	Homozygous	0.9938	LP
P38	PDE6B	NM:000283	c.2399del	p.Leu800Argfs*19	Heterozygous	N/A	LP
P49	IFT172	NM:015662	c.3228+13G>A	—	Homozygous	N/A	LP
P50	PDE6B	NM:000283	c.1108‐3C>G	—	Heterozygous	N/A	LP
P56	MYO7A	NM:000260.4	c.6539T>C	p.Leu2180Pro	Homozygous	0.9979	LP
P59	BBS7	NM:176824.3	c.849+1G>T	—	Homozygous	0.9958	LP
P64	ALMS1	NM:001378454	c.3154C>T	p.Gln1052*	Homozygous	0.9906	LP
P65	RS1	NM:000330	c.52+1G>C	—	Hemizygous	0.9935	LP
P67	TULP1	NM:003322	c.1199G>T	p.Arg400Leu	Homozygous	0.9986	LP
P72	ARL6	NM:001278293	c.276del	p.Phe92Leufs*9	Homozygous	N/A	LP
P78	USH2A	NM:206933	c.12574C>G	p.Arg 4192Gly	Homozygous	0.9931	LP
P81	PROM1	NM:006017	c.2118G>T	p.Gly706Gly	Homozygous	N/A	LP
P83	EYS	NM:001142800	c.1185–3C>A	—	Homozygous	N/A	LP
P84	RP1	NM:006269	c.109C>T	p.Arg37*	Homozygous	0.9979	LP
P84	HMCN1	NM:031935	c.12095G>A	p.Gly4032Asp	Homozygous	0.9944	LP
P86	MYO7A	NM:000260.4	c.4793T>C	p.Leu1598Pro	Homozygous	0.9992	LP
P89	IMPG2	NM:016247	c.1491del	p.Leu498Cysfs*15	Homozygous	N/A	LP
P90	PDE6B	NM:000283	c.1935C>G	p.Tyr645*	Homozygous	0.9959	LP
P92	IMPDH1	NM_000883	c.809T>C	p.Leu270Pro	Heterozygous	0.999	LP
P94	PDE6B	NM:000283	c.243del	p.Arg82Alafs*68	Homozygous	N/A	LP

*Note*: All novel variants were classified as pathogenic or likely pathogenic according to the American College of Medical Genetics and Genomics (ACMG) guidelines.

Abbreviations: LP, likely pathogenic; N/A, not available; P, pathogenic.

In our study, isolated RP was diagnosed in 28 patients (29.79%), 12 of whom had novel variants. RP is the most common type of IRDs. The worldwide prevalence is approximately 1 in 4000 [11]. AD, AR, X‐linked, or, more rarely, mitochondrial inheritance patterns are observed in RP patients [12]. Although the *RHO* gene is the first gene identified in RP patients, pathogenic variants have been identified in more than 100 genes involved in autosomal inherited RP. However, *RHO* is still the gene most associated with AD RP, accounting for more than 30% of cases, followed by *PRPH2*, *PRPF31*, and *RP1* [11]. We detected a heterozygous c.403C>T (p.Arg135Trp) pathogenic variant in the *RHO* gene in a patient with a history of RP and hearing loss (Patient 76). This variant has been previously reported in Chinese, Koreans, French, and Swedes [13, 14]. (p.Arg135Trp) pathogenic variant may disrupt ionic interactions and cause partial misfolding and reduced 11‐cis‐retinal binding ability of the mutant protein [15]. Together with several other reported pathogenic variants, including (p.Arg135Pro), (p.Arg135Leu), (p.Arg135Gly), and (p.Arg135Trp), this localization is thought to be a ‘hotspot’ pathogenic variant site [16].

Another IRDs subtype is Leber congenital amaurosis (LCA). LCA represents approximately 5% of all IRDs with a prevalence ranging from 1/30 000 to 1/81 000 [17]. LCA is the most severe form of IRDs seen in infancy. LCA was diagnosed in 10 of the patients included in the study (10.64%). A total of 12 pathogenic variants were found in 10 patients. While 8 of these were previously reported as pathogenic variants, 3 of them were reported for the first time in this study. There are 26 LCA‐related genes in the current database. Among these, *CEP290* (15%), *GUCY2D* (12%), *CRB1* (10%), and *RPE65* (8%) are the most frequently reported LCA genes with pathogenic variants [18]. In this study, pathogenic variants were found in the *RDH12* gene in 3 patients (30% of LCA) diagnosed with LCA and in the *CRB1* gene in 2 patients (20% of LCA). In the literature, nearly 120 pathogenic, likely pathogenic variants and VUSs have been reported in the *RDH12* gene, and 70% of these variants are missense [19]. Approximately 9%–17% of LCA cases have been associated with *CRB1* pathogenic variants [18]. Similar to the literature, a pathogenic variant in the *CRB1* gene was found in 20% of patients diagnosed with LCA in our study.

Stargardt disease (STGD) is the most common inherited macular dystrophy in both adults and children, with a prevalence of 1 in 8000–10 000 [20]. Stargardt disease type 1 (STGD1) has an AR mode of inheritance associated with disease‐causing pathogenic variants in the *ABCA4* gene. STGD1 was diagnosed in eight of the patients included in the study (8.5%). A total of 11 variants were found in 8 patients. While 10 of them were previously reported as pathogenic variants, 1 of them was previously unreported. To date, more than 1200 known disease‐causing variants at the *ABCA4* locus have been reported. Of these, 61% are missense variants and 23% are variants leading to protein termination [21, 22]. The variant in the *ABCA4* gene (p.Gly1961Glu), which causes the most common disease among the patients reported in the literature and which we detected in 6 patients, is the most common variant in people of East African origin and is found in ~10% of Somalis [23, 24]. In the study by Zernant et al., the patients carrying the (p.Gly1961Glu) variant in homozygote or compound heterozygote have a significantly different phenotype from patients carrying the other variant. While the mean age at onset in patients with ABCA4‐associated retinopathy was 19.7 years, the mean age at onset in ABCA4‐associated retinopathy cases carrying the (p.Gly1916Glu) allele was 22.7 years [21].

Among the 28 novel variants identified in our cohort, we classified them as likely pathogenic or pathogenic according to ACMG guidelines and showed genotype–phenotype consistency. For instance, novel variants in genes such as *ABCA4*, *RDH12*, and *CYP4V2* were observed in patients whose clinical features aligned with known disease presentations: Stargardt disease, Leber congenital amaurosis, and Bietti crystalline dystrophy, respectively. Although functional studies were not performed, the correlation between these variants and specific phenotypes strengthens their potential clinical relevance. Further functional validation and extended clinical follow‐up are necessary to better understand the impact of these novel findings and confirm their pathogenicity.

Four patients were diagnosed with BCD. Two of these 4 patients had the c.802‐8_810delinsGC variant, one had the c.1198C>T variant, and one had the c.414‐1G>T variant in the *CYP4V2* gene. The c.802‐8_810delinsGC variant is the pathogenic variant known to be common in Asian populations [25]. Genotype–phenotype correlation has not yet been established in studies conducted on BCD patients [26]. However, in two different studies conducted by Lai and Halford, it was observed that patients with the c.802‐8_810delinsGC pathogenic variant had more severe phenotypes in electrophysiological evaluations [27, 28].

US was diagnosed in 7 (8%) of the patients included in the study. Pathogenic variants were detected in the *USH2A* gene in 6 of them and in the *MYO7A* gene in 2 of them. In patient 2, a homozygous c.12067‐2A>G variant was detected in the *USH2A* gene. The same variant was heterozygous in patient number 7. This variant was first reported in 2008 by Auslender et al. in a study conducted in Israel on patients of non‐Ashkenazi origin [29]. In the same study, it was reported that the A>G change in the conserved acceptor site may lead to a splicing defect [29].

Our findings are also in line with previous IRD studies conducted in Türkiye. In a recent study by Başdemirci et al., a diagnostic rate of 92.9% was reported using whole‐exome sequencing in 28 unrelated Turkish patients, with several novel variants identified in genes such as *RDH12*, *TULP1*, and *MERTK* [30]. Although the diagnostic rate was 92.9%, which is considerably higher than in our study and previous reports in the literature, 39.2% of the genetically diagnosed patients in that study carried variants classified as variants of uncertain significance (VUS). Pathogenic or likely pathogenic variants were reported in 53.6% of the cases [30]. Another large‐scale cohort involving 50 Turkish IRD patients found a diagnostic yield of 58%, which highlights the variability in detection rates depending on methodology, panel content, and population characteristics [31]. A broader regional study including Turkish and Syrian patients identified 175 different pathogenic variations, 58 of which were novel, further illustrating the genetic heterogeneity of IRDs in this geographic region [32]. In our study, we achieved a diagnostic rate of 74% using a targeted 141‐gene IRD panel and identified 28 novel variants, particularly in *ABCA4*, *CRB1*, and *RDH12*. These findings support the clinical utility of NGS in IRDs and emphasize the genetic heterogeneity observed in the Turkish population. Additionally, our contribution of novel variants further expands the known mutational spectrum of IRDs in this region.

A limitation of this study is the inability to perform segregation analysis for some variants identified in probands and to screen relatives with similar family histories, due to the deceased status of certain family members. Also, not all genes associated with IRDs could be analyzed. More comprehensive studies, including all genes related to IRDs in patients with undiagnosed IRDs, are needed.

Although we did not perform formal hypothesis testing or power analysis, the relatively small sample size may limit the interpretability of subgroup findings and the ability to detect rare genotype–phenotype relationships. The observed distribution patterns are consistent with the literature; however, larger multicenter studies are needed to validate and generalize these observations.

In addition, although our NGS panel was designed to cover the exonic and splice‐site regions of 141 IRD‐associated genes, it may fail to detect pathogenic variants located in deep intronic regions or other regulatory non‐coding sequences not targeted by the panel. This may explain why some patients remain without a molecular diagnosis.

## 5 Conclusion

Early molecular diagnosis in IRD patients is important for both disease management and the evaluation of appropriate treatment options. Especially with the widespread use of gene replacement therapies, it has become more important to determine the underlying genetic etiology in patients. In conclusion, a 74% diagnostic rate was achieved, demonstrating the high efficacy of NGS‐based gene panel analysis for IRDs. Also, our study contributes to the literature for the spectrum of IRD‐related variants in the literature with 28 novel variants.

## Author Contributions

Senol Demir, Bilgen Bilge Geckinli, and Esra Arslan Ates contributed to the design of the manuscript. Tugba Kose and Ahmet Arman performed data analysis. Senol Demir and Bilgen Bilge Geckinli wrote the manuscript. Orkun Sevik and Bengisu Sozer conducted the patients physical examination. Ahmet Arman, Ozlem Sahin, and Esra Arslan Ates critically reviewed the manuscript. All authors contributed to the article.

## Disclosure

The authors alone are responsible for the content and writing of this article.

## Ethics Statement

For this research, the Ethics Committee of Marmara University approval was obtained with the decision of the Board of Ethics Committee numbered 09.2023.876.

## Conflicts of Interest

The authors declare no conflicts of interest.

## Peer Review

The peer review history for this article is available at https://www.webofscience.com/api/gateway/wos/peer‐review/10.1111/cge.14769.

## Supporting information


**Table S1.** Genes included in the 141‐gene IRD panel.

## Data Availability

The data that support the findings of this study are available from the corresponding author upon reasonable request.

## References

[1] J. A. Sahel , K. Marazova , and I. Audo , “Clinical Characteristics and Current Therapies for Inherited Retinal Degenerations,” Cold Spring Harbor Perspectives in Medicine 5, no. 2 (2014): a017111. 25324231 10.1101/cshperspect.a017111 PMC4315917

[2] Y. Tatour and T. Ben‐Yosef , “Syndromic Inherited Retinal Diseases: Genetic, Clinical and Diagnostic Aspects,” Diagnostics (Basel) 10, no. 10 (2020): 779. 33023209 10.3390/diagnostics10100779 PMC7600643

[3] J. M. Schwarz , D. N. Cooper , M. Schuelke , and D. Seelow , “MutationTaster2: Mutation Prediction for the Deep‐Sequencing Age,” Nature Methods 11, no. 4 (2014): 361–362. 24681721 10.1038/nmeth.2890

[4] F. O. Desmet , D. Hamroun , M. Lalande , G. Collod‐Béroud , M. Claustres , and C. Béroud , “Human Splicing Finder: An Online Bioinformatics Tool to Predict Splicing Signals,” Nucleic Acids Research 37, no. 9 (2009): e67. 19339519 10.1093/nar/gkp215 PMC2685110

[5] S. Richards , N. Aziz , S. Bale , et al., “Standards and Guidelines for the Interpretation of Sequence Variants: A Joint Consensus Recommendation of the American College of Medical Genetics and Genomics and the Association for Molecular Pathology,” Genetics in Medicine 17, no. 5 (2015): 405–424. 25741868 10.1038/gim.2015.30 PMC4544753

[6] C. Kopanos , V. Tsiolkas , A. Kouris , et al., “VarSome: The Human Genomic Variant Search Engine,” Bioinformatics 35, no. 11 (2019): 1978–1980. 30376034 10.1093/bioinformatics/bty897 PMC6546127

[7] V. G. Peter , K. Kaminska , C. Santos , et al., “The First Genetic Landscape of Inherited Retinal Dystrophies in Portuguese Patients Identifies Recurrent Homozygous Mutations as a Frequent Cause of Pathogenesis,” PNAS Nexus 2, no. 3 (2023): pgad043. 36909829 10.1093/pnasnexus/pgad043 PMC10003751

[8] C. Villanueva‐Mendoza , M. Tuson , D. Apam‐Garduño , et al., “The Genetic Landscape of Inherited Retinal Diseases in a Mexican Cohort: Genes, Mutations and Phenotypes,” Genes (Basel) 12, no. 11 (2021): 1824. 34828430 10.3390/genes12111824 PMC8624043

[9] B. Ozguc Caliskan , K. Uslu , N. Sinim Kahraman , K. Erkilic , A. Oner , and M. Dundar , “Beyond the Phenotype: Exploring Inherited Retinal Diseases With Targeted Next‐Generation Sequencing in a Turkish Cohort,” Clinical Genetics 106 (2024): 258–266. 38576124 10.1111/cge.14529

[10] M. Karali , F. Testa , V. di Iorio , et al., “Genetic Epidemiology of Inherited Retinal Diseases in a Large Patient Cohort Followed at a Single Center in Italy,” Scientific Reports 12, no. 1 (2022): 20815. 36460718 10.1038/s41598-022-24636-1 PMC9718770

[11] S. K. Verbakel , R. A. C. van Huet , C. J. F. Boon , et al., “Non‐Syndromic Retinitis Pigmentosa,” Progress in Retinal and Eye Research 66 (2018): 157–186. 29597005 10.1016/j.preteyeres.2018.03.005

[12] C. Rivolta , D. Sharon , M. DeAngelis , and T. P. Dryja , “Retinitis Pigmentosa and Allied Diseases: Numerous Diseases, Genes, and Inheritance Patterns,” Human Molecular Genetics 11, no. 10 (2002): 1219–1227. 12015282 10.1093/hmg/11.10.1219

[13] C. Kim , K. J. Kim , J. Bok , et al., “Microarray‐Based Mutation Detection and Phenotypic Characterization in Korean Patients With Retinitis Pigmentosa,” Molecular Vision 18 (2012): 2398–2410. 23049240 PMC3462597

[14] W. Abdulridha‐Aboud , U. Kjellström , S. Andréasson , and V. Ponjavic , “Characterization of Macular Structure and Function in Two Swedish Families With Genetically Identified Autosomal Dominant Retinitis Pigmentosa,” Molecular Vision 22 (2016): 362–373. 27212874 PMC4860447

[15] W. B. Ou , T. Yi , J. M. Kim , and H. G. Khorana , “The Roles of Transmembrane Domain Helix‐III During Rhodopsin Photoactivation,” PLoS One 6, no. 2 (2011): e17398. 21364764 10.1371/journal.pone.0017398 PMC3045455

[16] Y. Wu , Y. Guo , J. Yi , et al., “Heterozygous RHO p.R135W Missense Mutation in a Large Han‐Chinese Family With Retinitis Pigmentosa and Different Refractive Errors,” Bioscience Reports 39, no. 7 (2019): BSR20182198. 31239368 10.1042/BSR20182198 PMC6629948

[17] C. H. Huang , C. M. Yang , C. H. Yang , Y. C. Hou , and T. C. Chen , “Leber's Congenital Amaurosis: Current Concepts of Genotype–Phenotype Correlations,” Genes (Basel) 12, no. 8 (2021): 1261. 34440435 10.3390/genes12081261 PMC8392113

[18] N. Kumaran , A. T. Moore , R. G. Weleber , and M. Michaelides , “Leber Congenital Amaurosis/Early‐Onset Severe Retinal Dystrophy: Clinical Features, Molecular Genetics and Therapeutic Interventions,” British Journal of Ophthalmology 101, no. 9 (2017): 1147–1154. 28689169 10.1136/bjophthalmol-2016-309975 PMC5574398

[19] A. T. Fahim and D. A. Thompson , “Natural History and Genotype‐Phenotype Correlations in RDH12‐Associated Retinal Degeneration,” Advances in Experimental Medicine and Biology 1185 (2019): 209–213. 31884613 10.1007/978-3-030-27378-1_34 PMC7065034

[20] M. Michaelides , D. M. Hunt , and A. T. Moore , “The Genetics of Inherited Macular Dystrophies,” Journal of Medical Genetics 40, no. 9 (2003): 641–650. 12960208 10.1136/jmg.40.9.641 PMC1735576

[21] J. Zernant , W. Lee , F. T. Collison , et al., “Frequent Hypomorphic Alleles Account for a Significant Fraction of ABCA4 Disease and Distinguish It From Age‐Related Macular Degeneration,” Journal of Medical Genetics 54, no. 6 (2017): 404–412. 28446513 10.1136/jmedgenet-2017-104540 PMC5786429

[22] N. Zhang , Y. Tsybovsky , A. V. Kolesnikov , et al., “Protein Misfolding and the Pathogenesis of ABCA4‐Associated Retinal Degenerations,” Human Molecular Genetics 24, no. 11 (2015): 3220–3237. 25712131 10.1093/hmg/ddv073 PMC4424957

[23] T. R. Burke , S. Yzer , J. Zernant , R. T. Smith , S. H. Tsang , and R. Allikmets , “Abnormality in the External Limiting Membrane in Early Stargardt Disease,” Ophthalmic Genetics 34, no. 1–2 (2013): 75–77. 22871184 10.3109/13816810.2012.707271 PMC4115808

[24] R. H. Guymer , E. Héon , A. J. Lotery , et al., “Variation of Codons 1961 and 2177 of the Stargardt Disease Gene Is Not Associated With Age‐Related Macular Degeneration,” Archives of Ophthalmology 119, no. 5 (2001): 745–751. 11346402 10.1001/archopht.119.5.745

[25] D. S. Ng , T. Y. Lai , T. K. Ng , and C. P. Pang , “Genetics of Bietti Crystalline Dystrophy,” Asia‐Pacific Journal of Ophthalmology (Philadelphia, PA) 5, no. 4 (2016): 245–252. 27228076 10.1097/APO.0000000000000209

[26] L. W. Chan , Y. C. Sung , D. C. Wu , et al., “Predicted Protein Structure Variations Indicate the Clinical Presentation of Cyp4v2‐Related Bietti Crystalline Dystrophy,” Retina 42, no. 4 (2022): 797–806. 34923510 10.1097/IAE.0000000000003381

[27] T. Y. Lai , T. K. Ng , P. O. Tam , et al., “Genotype Phenotype Analysis of Bietti's Crystalline Dystrophy in Patients With CYP4V2 Mutations,” Investigative Ophthalmology & Visual Science 48, no. 11 (2007): 5212–5220. 17962476 10.1167/iovs.07-0660

[28] S. Halford , G. Liew , D. S. Mackay , et al., “Detailed Phenotypic and Genotypic Characterization of Bietti Crystalline Dystrophy,” Ophthalmology 121, no. 6 (2014): 1174–1184. 24480711 10.1016/j.ophtha.2013.11.042

[29] N. Auslender , D. Bandah , L. Rizel , et al., “Four USH2A Founder Mutations Underlie the Majority of Usher Syndrome Type 2 Cases Among Non‐Ashkenazi Jews,” Genetic Testing 12, no. 2 (2008): 289–294. 18452394 10.1089/gte.2007.0107

[30] M. Basdemirci and H. Kocak Eker , “Whole‐Exome Sequencing in Turkish Patients With Inherited Retinal Dystrophies Reveals Novel Variants in Ten Genes,” Molecular Syndromology 15, no. 3 (2024): 202–210. 38841332 10.1159/000535590 PMC11149968

[31] C. Yavas , Y. E. Arvas , M. Dogan , et al., “Revealing Molecular Diagnosis With Whole Exome Sequencing in Patients With Inherited Retinal Disorders,” Clinical Genetics 108, no. 1 (2025): 14–21. 39865314 10.1111/cge.14708 PMC12136866

[32] I. Sahin , H. B. Erdem , T. Bahsi , and H. Saat , “Expanding the Genotype‐Phenotype Correlations and Mutational Spectrum in Inherited Retinal Diseases: Novel and Recurrent Mutations,” Cureus 16, no. 2 (2024): e53742. 38465142 10.7759/cureus.53742 PMC10920963

