# Supplementary material for: Decoding the Genetic Puzzle of Inherited Retinal Dystrophies: Novel Insights From a Turkish Cohort
Source: Clin Genet. 2025 May 15;108(5):532–52. doi: 10.1111/cge.14769 (PMC12501730; doi:10.1111/cge.14769)
Supplement: Supplementary file 1 — Table S1. Genes included in the 141‐gene IRD panel. [file CGE-108-532-s001.docx]

| ABCA4 | GPR179 | PITPNM3 |
| --- | --- | --- |
| ABHD12 | GRK1 | POMGNT1 |
| ADAM9 | GRM6 | PRCD |
| AHI1 | GUCA1A | PROM1 |
| AHR | GUCA1B | PRPF3 |
| AIPL1 | GUCY2D | PRPF31 |
| ALMS1 | HGSNAT | PRPF6 |
| ARL3 | HK1 | PRPF8 |
| ARL6 | IDH3B | PRPH2 |
| ATF6 | IFT140 | RAX2 |
| BBS1 | IFT172 | RBP3 |
| BBS10 | IFT43 | RD3 |
| BBS12 | IFT88 | RDH12 |
| BBS2 | IL10 | RDH5 |
| BBS4 | IL12RB2 | RGR |
| BBS5 | IL23R | RHO |
| BBS7 | IMPDH1 | RIMS1 |
| BBS9 | IMPG2 | RLBP1 |
| BEST1 | IQCB1 | ROM1 |
| C8orf37 | KCNJ13 | RP1 |
| CA4 | KCNV2 | RP1L1 |
| CABP4 | KLHL7 | RP2 |
| CACNA1F | LCA5 | RP9 |
| CACNA2D4 | LRAT | RPE65 |
| CDH3 | LRIT3 | RPGR |
| CDHR1 | LZTFL1 | RPGRIP1 |
| CEP290 | MAK | RPGRIP1L |
| CERKL | MEFV | SAG |
| CLRN1 | MERTK | SDCCAG8 |
| CNGA1 | MFRP | SEMA4A |
| CNGA3 | MKKS | SLC19A2 |
| CNGB1 | MKS1 | SLC24A1 |
| CNGB3 | NEUROD1 | SNRNP200 |
| CNNM4 | NMNAT1 | SPATA7 |
| CRB1 | NOD2 | STAT4 |
| CRX | NPHP1 | TLR4 |
| CYP4V2 | NR2E3 | TOPORS |
| DHDDS | NRL | TRIM32 |
| ELOVL4 | NYX | TRPM1 |
| EYS | OFD1 | TTC8 |
| FAM161A | PANK2 | TUB |
| FLVCR1 | PCARE | TULP1 |
| FSCN2 | PDE6A | UNC119 |
| GDF6 | PDE6B | USH2A |
| GGCX | PDE6C | WDPCP |
| GNAT1 | PDE6G | WDR19 |
| GNAT2 | PDE6H | ZNF513 |

# **Table S1. Genes Included in the 141-Gene IRD Panel**
